# Prefrontal activation in response to a plantar contact task under open and closed eye conditions in patients with cerebral infarction

**DOI:** 10.3389/fnins.2023.1255354

**Published:** 2023-09-12

**Authors:** Zhi-Quan Yang, Meng-Fan Wei, Jia-Ning Xi

**Affiliations:** ^1^Beijing Rehabilitation Hospital, Capital Medical University, Beijing, China; ^2^Beijing Zhongguancun Hospital/Zhongguancun Hospital, Chinese Academy of Sciences, Beijing, China

**Keywords:** fNIRS, plantar contact task, cerebral infarction, dorsolateral prefrontal lobe, rehabilitative

## Abstract

**Objective:**

This study investigates the effect of a bilateral (paralyzed side, healthy side) plantar contact task on dorsolateral prefrontal activation in patients recovering from cerebral infarction under open and closed eye conditions.

**Methods:**

We selected 10 patients with cerebral infarction, admitted to the neurorehabilitation center of Beijing Rehabilitation Hospital, affiliated with Capital Medical University, from January 2019 to July 2020, who met our established criteria. Under open-eye and closed-eye conditions, the paralyzed and healthy sides performed the plantar contact tasks separately. The dorsolateral prefrontal region was monitored simultaneously with functional near-infrared spectroscopy (fNIRS), and activation was analyzed according to the curve-type changes of oxyhemoglobin and deoxyhemoglobin changes in the dorsolateral prefrontal cortex with 560 near-infrared monitoring channels.

**Results:**

After stratifying the data based on the eyes-open and eyes-closed conditions, some degree of heterogeneity was observed between the layers. Under the eyes-closed condition, the Pearson χ^2^ was 0.142, with a *p* value of 0.706, indicating no significant impact of the eyes-closed condition on the activation of the dorsolateral prefrontal cortex during the plantar task, whether performed on the paralyzed or the healthy side.

In contrast, the Pearson χ^2^ value was 15.15 for the eyes-open condition, with a *p* value of 0.002. This suggests that carrying out the plantar task, either on the paralyzed or the healthy side, with eyes open significantly influenced the activation of the dorsolateral prefrontal cortex. Furthermore, activation of the dorsolateral prefrontal cortex was 1.55 times higher when the task was executed with the paralyzed side compared to the healthy side. This implies that the paralyzed side was more likely to activate the dorsolateral prefrontal lobe when performing the plantar contact task under eyes-open conditions.

**Conclusion:**

Observations via fNIRS revealed that the plantar contact task elicited dorsolateral prefrontal cortex activation. Moreover, the activation effect was intensified when performed on the paralyzed side under eyes-open conditions. Therapeutic methods that leverage these findings—namely cognitive-motor therapies that promote the recovery of motor functions by activating cognitive control brain regions via perception (information construction)—may hold promise.

## 1. Introduction

Stroke is a disease caused by impaired cerebral blood circulation with a high rate of disability (Maenza et al., 2020). Studies indicate that approximately 90% of individuals recovering from stroke experience varying levels of functional impairment, with walking dysfunction among the most prominent (Hobbs and Artemiadis, 2020). Hemiparesis (Bogousslavsky et al., 1996), the most common functional deficit among patients with cerebral infarction, often results in patients’ inability to walk independently, necessitating reliance on assistance from others or wheelchair use for mobility. Even those who regained independent walking face challenges such as decreased foot contouring ability, compromised lower limb stability, and diminished walking efficiency due to foot drop during the swing phase and foot lateral edge contact at the end of the stance phase. These complications contribute to reduced ambulatory capabilities and a significantly increased fall risk. This severely affects patients’ mobility, safety, and quality of life, acting as a major barrier for stroke survivors with hemiplegia to reintegrate into their families and society.

Regaining walking ability is a major task for motor function and activities of daily living (ADL) rehabilitation in hemiplegic stroke patients. In clinical practice, cognitive-motor therapy, which emphasizes perceptual input and cognitive-driven movement, has been increasingly practiced clinically in improving walking ability in patients with cerebral infarction, especially in tasks requiring attention and processing speed, such as multitasking and gait adaptation tasks (Montero-Odasso et al., 2012). Some studies demonstrated the importance of cortical function for locomotion, as well as a greater emphasis on some methods to improve motor function by activating cognitive processes in the cortex (Fritz et al., 2015; Pothier et al., 2018; Hazra et al., 2022). In addition, cognitive-motor training, in which a cognitive task is performed alongside motor training, can more effectively strengthen the functional brain network connections between motor-cognitive brain areas and facilitate the activation of the cerebral cortex, thus promoting brain functional network remodeling and improving the patient’s functional impairment (Caetano et al., 2017; Pang et al., 2018). However, the mechanism of action may involve brain functional remodeling, motor relearning, and neural facilitation, but its neurophysiological mechanism is not established.

Hence, we designed a plantar contact task based on the therapeutic principles of cognitive-motor therapy and used a portable fNIRS technique to monitor its effects on dorsolateral prefrontal activation during the treatment to analyze and explore the possible mechanisms of perception (constructing information) to promote motor function recovery. We hypothesize that cognitive-motor therapy can promote the recovery of motor function in cerebral infarction patients by activating cognitive control of brain areas through perceptual haptics.

## 2. Research subjects and methods

### 2.1. Research subjects

We selected 10 patients with cerebral infarction, admitted to the neurorehabilitation center of Beijing Rehabilitation Hospital, affiliated with Capital Medical University, from January 2019 to July 2020, who met our established criteria. The inclusion criteria included: ① Patients who met the diagnostic criteria of cerebral infarction formulated by the Fourth Academic Conference on Cerebrovascular Diseases (Neurology CSo, 2015) and confirmed as the first onset by cranial CT or MRI examination; ② Aged between 18 and 65 years; ③ Duration of disease less than 6 months, stable vital signs, clear consciousness, and ability to follow instructions; ④ Absence of serious acute or chronic heart valve disease, cardiomyopathy, frequent recent attacks of angina pectoris, unstable angina pectoris, or other organic heart diseases; ⑤ The ability to maintain an independent sitting position with knees capable of more than 90 degrees flexion and feet capable of sliding backward; ⑥ Normal vestibular system function and proprioception.

The exclusion criteria included: ① Pregnant or lactating women; ② Those with involuntary twitching, tremor, or other severe organic diseases and neurological diseases, who were unable to cooperate with the completion of the examination; ③ Aphasia; ④ Mental impairment, hearing impairment, comprehension impairment, or severe cognitive impairment; ⑤ History of orthopedic surgery, hip dislocation, unhealed fractures, or severe osteoporosis; ⑥ Presence of scoliosis and other spinal deformities; ⑦ Malignant tumors, bleeding tendency; ⑧ Patients with deteriorating conditions, with new infarct foci, or cerebral hemorrhage.

The study was approved by the Ethics Committee of Beijing Rehabilitation Hospital, Capital Medical University, and all study subjects provided written informed consent.

### 2.2. Research methods

#### 2.2.1. Apparatus for fNIRS test

**Figure 1:**
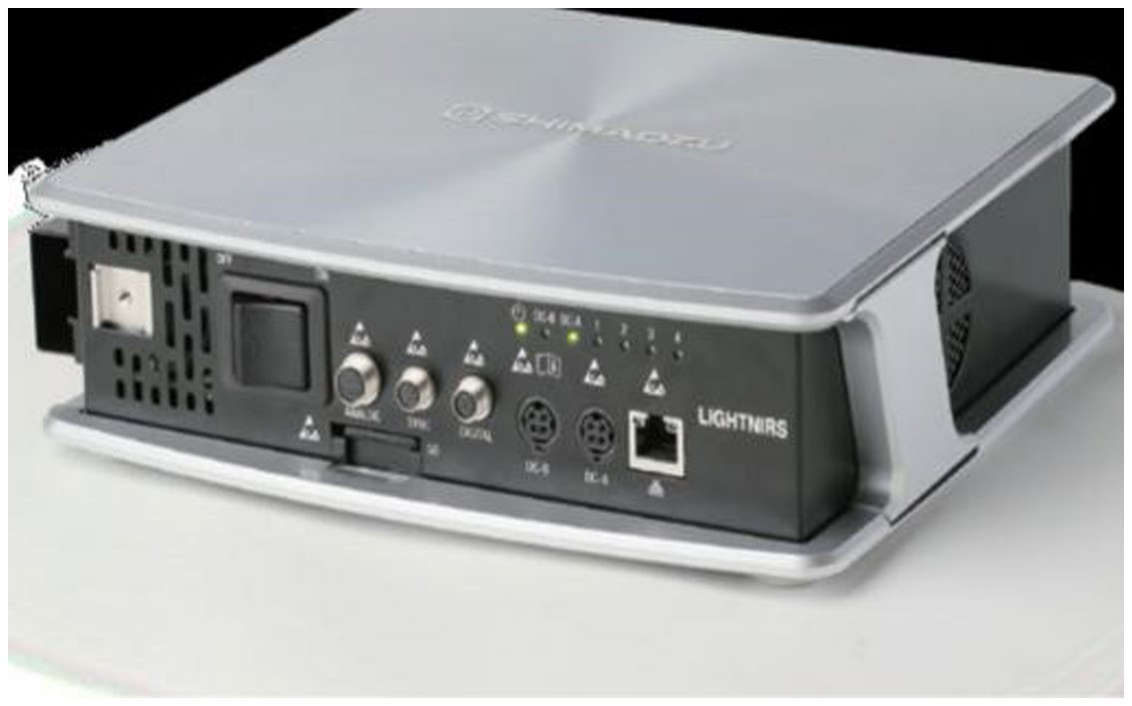
Portable fNIRS brain imaging system.

This study used a portable fNIRS brain imaging system, from Shimadzu, Japan (Figure 1). This system employs a 3-wavelength (780 nm, 805 nm, and 830 nm) absorbance meter algorithm to measure the changes in oxygenated hemoglobin (Oxy-Hb), deoxygenated hemoglobin (Deoxy-Hb), and total hemoglobin concentrations. The light source of this system is a 3-wavelength near-infrared (NIR) semiconductor laser, and the detector is an avalanche photodiode. The device is powered by a 15 V AC adapter or a lithium battery, and the external output includes a 3-bit digital signal and a 10-bit analog signal. The dimensions are W253 × D222 × H68 mm (excluding protruding parts), and the weight is approximately 1,600 g (excluding the computer, battery, and fiber).

**Figure 2:**
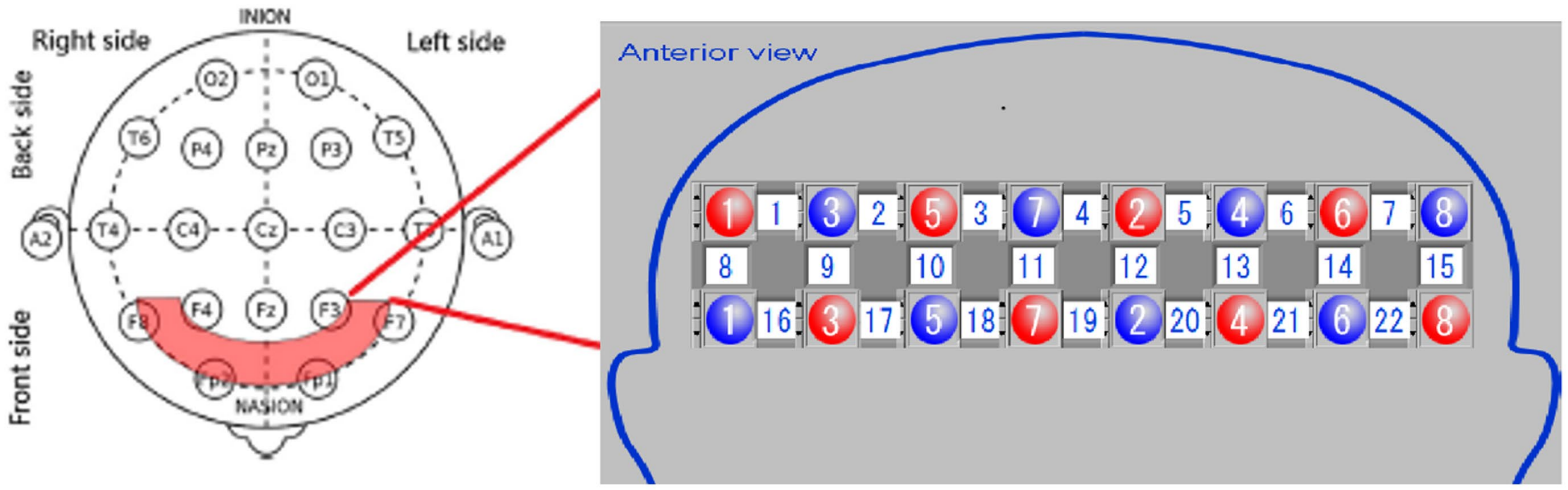
Placement of optical poles and test area.

The equipment comprises an optical fiber, a probe, fNIRS detection equipment, and a laptop computer. The probe is color-coded, with red representing an integrated LED light source head combining 780, 805, and 830 nm wavelengths, while blue indicates the near-infrared light detection head. The probe converts the NIR light signals detected during the experiment into electrical signals for further processing in the computer. The testing machine communicates wirelessly with the computer. During testing, the light source probe and detection receiving probe are secured using fiber optic cap jacks, forming a 2×8 array with 22 sampling channels (Figure 2). Each testing area, or sampling channel, is determined by the area between every two adjacent light source heads and receiving heads.

#### 2.2.2. Test procedure

The experimental conditions were set to an ambient temperature of 15–30°C, with a temperature variation within 5°C/h, relative humidity of 45–85% (ensuring no dew or ice formation), and air pressure between 700 and 1,060 hPa.

The subjects were dressed in loose clothing and barefoot, though socks were allowed. They wore portable fNIRS head caps. Using continuous wave (CW) technology mode, we monitored the dorsolateral prefrontal cortex (PFC) with a time resolution of 0.075 s and a 2.0–3.0 cm depth to measure the concentrations of Oxy-Hb and Deoxy-Hb.

For optode placement, 8 emitting optodes (red dots) and 8 receiving optodes (blue dots) were positioned on the subject’s dorsolateral prefrontal lobe, maintaining a 3 cm spacing. The detection area was the region between the emitting and receiving optodes, constituting a total of 22 detection channels. We focused on the 14 channels covering the dorsolateral prefrontal lobe. The frontopolar midline point (Fp) was determined according to the international 10–20 electrode configuration method, ensuring the central emitting optode was positioned on the Fp point (Figure 2). Before the experiment, we cleared the patient’s hair from the scalp area where the optodes were placed. Following installation, an instrument self-test was performed to confirm the smooth optical path.

**Figure 3:**
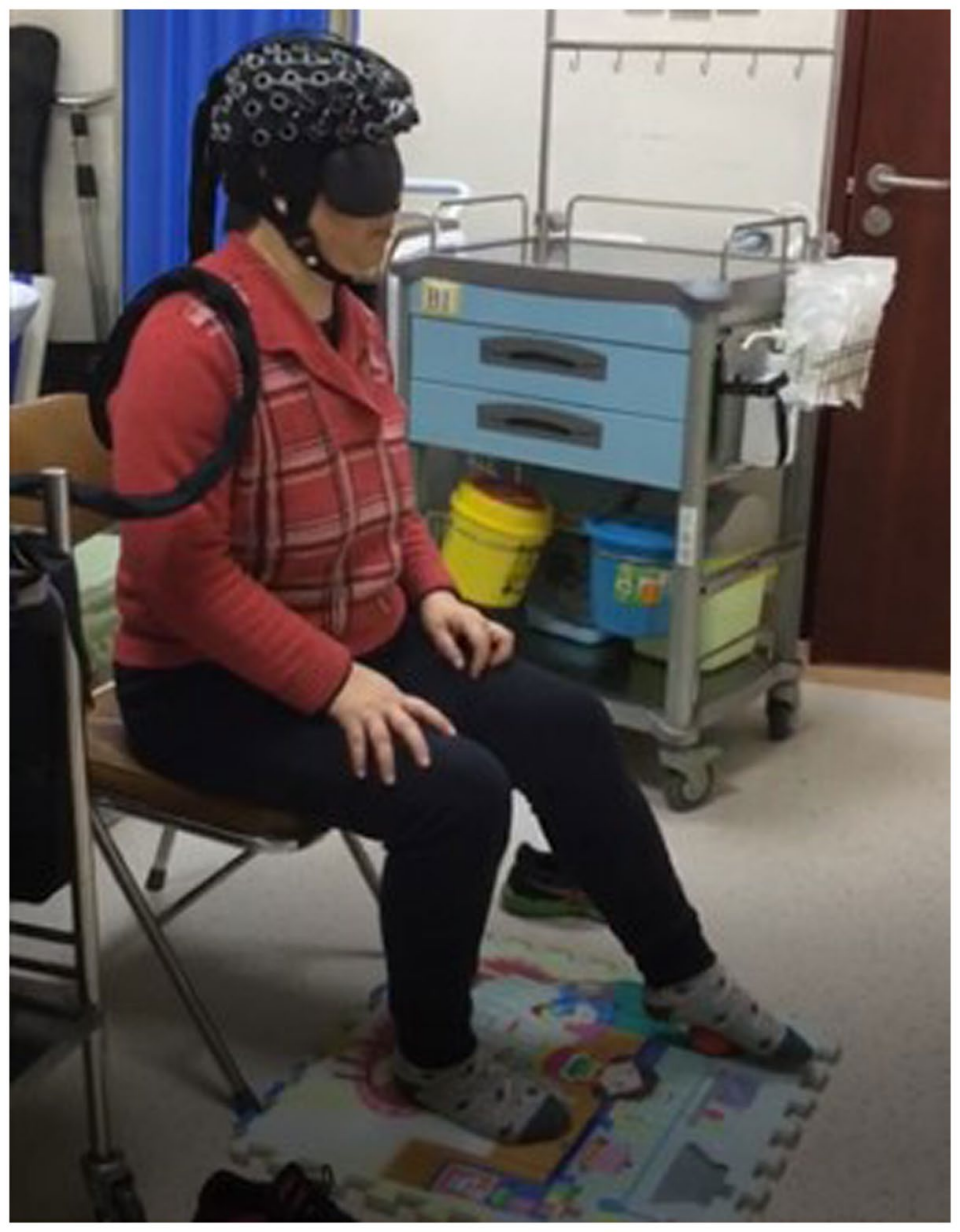
Plantar contact task (the patient was asked to perform a plantar contact task, moving the ball from heel to toe and vice versa at a controlled speed, flexing and extending the knee joint, and feeling the trajectory changes of the ball under their foot).

**Figure 4:**
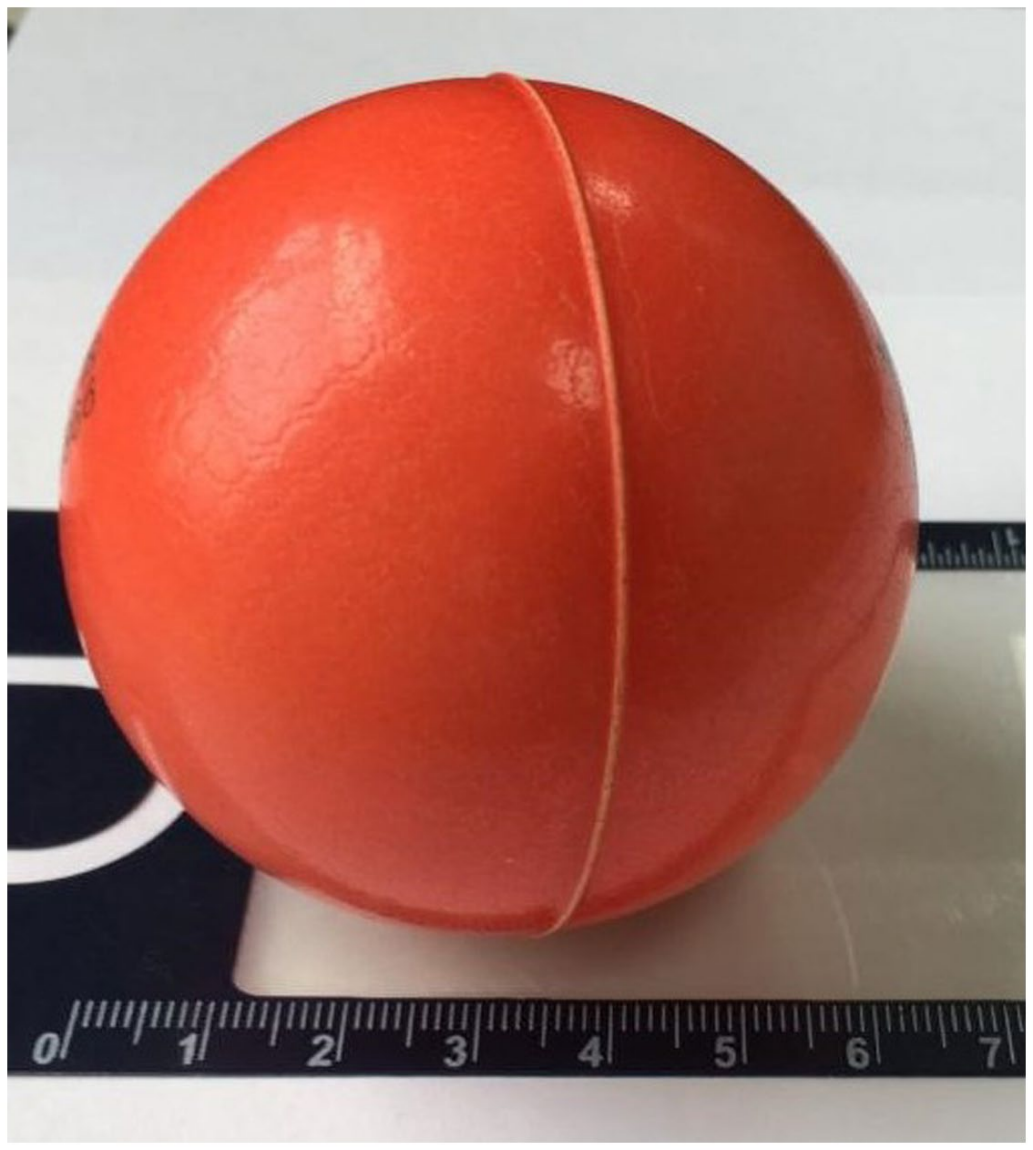
Red soft elastic sphere (diameter 6 cm).

The procedure involved (1) the patient relaxing, sitting, and stepping on a small 6 cm diameter red elastic ball (Figures 3, 4) under Light NIRS monitoring; (2) while seated, the patient was asked to perform a plantar contact task, moving the ball from heel to toe and vice versa at a controlled speed, flexing and extending the knee joint, and feeling the trajectory changes of the ball under their foot; (3) the activation of the dorsolateral prefrontal lobe was tested under eyes-open and eyes-closed conditions, with both the paralyzed and healthy side of the lower limb performing the task.

During the procedure, a professional staff member was present. If a patient experienced pain or the ball slid from under their foot, the test was paused, adjustments were made for comfort or to restore the ball’s position, and then the test resumed.

#### 2.2.3. Observed indicators

**Figure 5:**
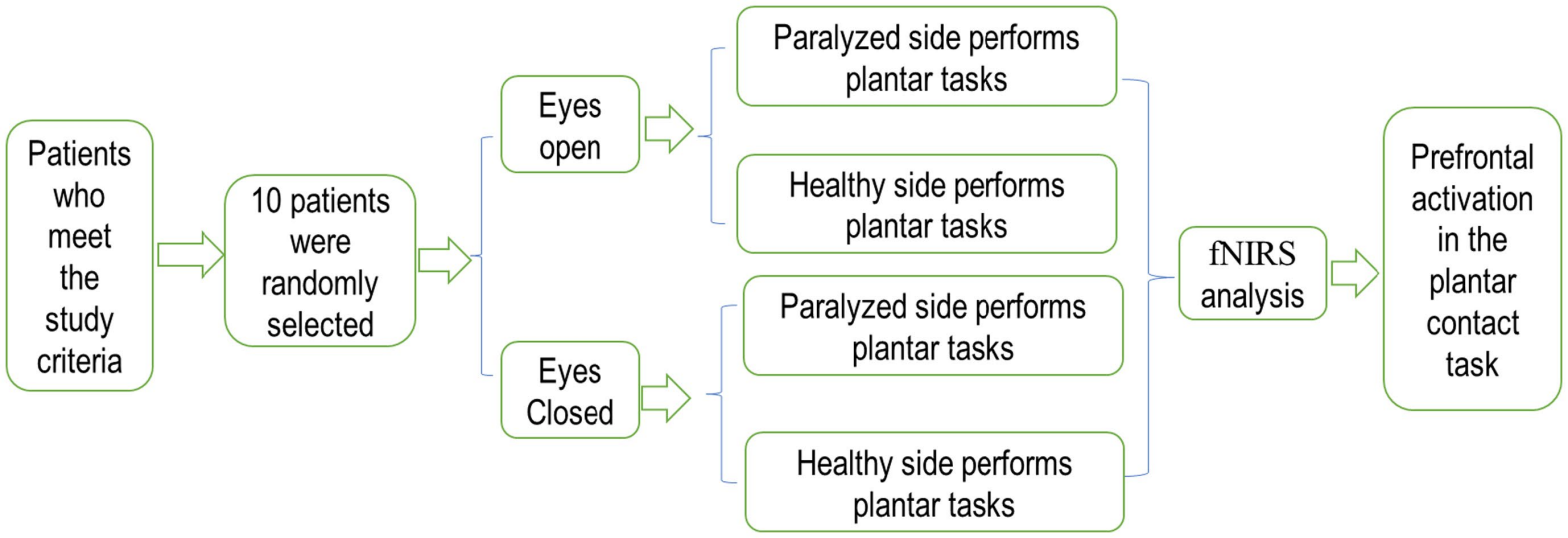
The study protocol.

The plantar contact task designed for this trial was based on cognitive-motor therapy principles designed to promote improved motor function in the affected knee and ankle by reconstituting the cognitive process of the plantaris minor (Cabral et al., 2022). Because of the limited number of optodes lined up for fixation and test optodes in the fiber optic cap used for testing, the cortical domain of the dorsolateral prefrontal area, a key brain region associated with many higher cognitive functions, was selected for fNIRS monitoring. The Brodmann areas were used as the basis for the localization of prefrontal cortical areas, while the 10–20 systematic map, frequently used in EEG for brain functional area localization, aided in determining the distribution of each channel at different regions of interest (ROIs). Among the channels, the right dorsolateral prefrontal cortex (RDPFC) was primarily covered by channels 1, 2, 8, 9, 10, 16, and 17. In contrast, channels 6, 7, 13, 14, 15, 21, and 22 mainly focused on the left dorsolateral prefrontal cortex (LDPFC). Subjects in the experiment were tested for a total of four separate testing sessions, included the plantar contact task on the paralyzed side of the eyes-open condition, the healthy side of the eyes-open condition, the paralyzed side of the eyes-closed condition, and the healthy side of the eyes-closed condition, respectively (Figure 5). In this experiment, our primary attention was on 560 channels (ch; 14ch/case × 10 cases/session × 4 sessions = 560ch).

#### 2.2.4. Methods for analysis of brain activation

**Figure 6:**
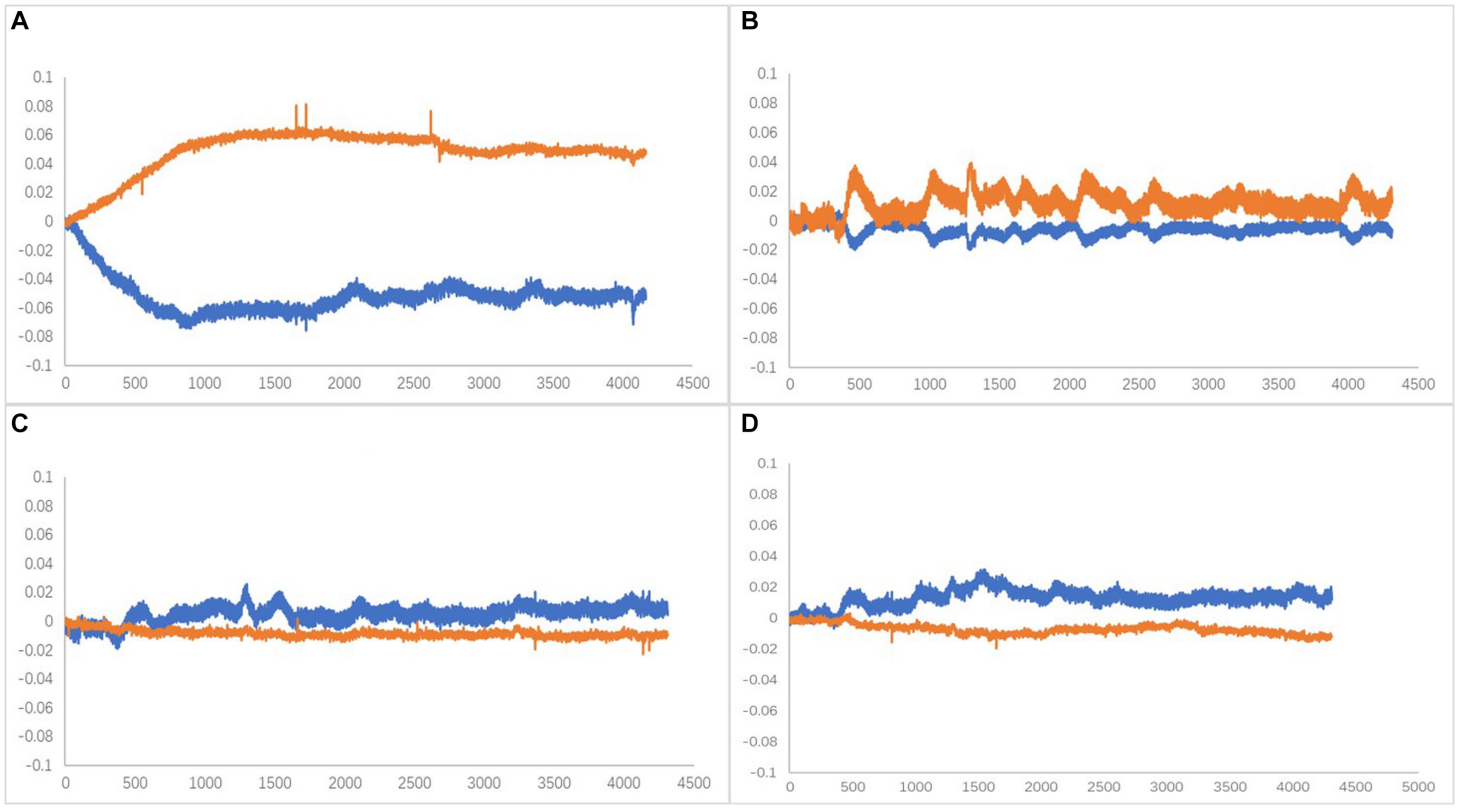
**(A)** Type A, Oxy-Hb upstream separated (activated). **(B)** Type B, Companion Oxy-Hb up (activated). **(C)** Type C, Companion Oxy-Hb downstream. **(D)** Type D, Oxy-Hb downstream separated. X-axis is time (ms),Y-axis is compound concentration (mmol/L).

Oxy-Hb has been identified as the most sensitive indicator of regional cerebral blood flow among fNIRS signals (Hoshi et al., 1985). Cognitive-related tasks are known to induce changes in brain rheological parameters, serving as a stable parameter for brain oxygenation. Activation of brain regions leads to dilation of local blood supply, increasing blood flow by approximately 30–50% and boosting blood oxygen consumption by around 5%. These changes prompt a hemodynamic response that often results in elevated levels of blood Oxy-Hb and decreased Deoxy-Hb levels (Suto et al., 2004; Nakahachi et al., 2008). Therefore, in this study, the relationship between the changes in the Oxy-Hb and Deoxy-Hb curves during the time phase of performing the plantar contact task was divided into four types (Figure 6), and subsequent statistical analyses were performed with the characteristic curve changes of all channels.

#### 2.2.5. Statistical analysis

SPSS 26.0 software was used to complete the data processing. The total number of channels in this experiment was 560ch (14ch/case × 10 cases/ session × 4 sessions = 560ch), and the effect on the activation of each channel in the prefrontal lobe was hierarchical listing information in the open-eye condition, paralyzed side versus healthy side, and the statistical inference of its independence test was made by stratified χ^2^ test. *p* < 0.05, statistically significant.

## 3. Results

### 3.1. Activation of each channel in the dorsolateral prefrontal lobe

The dorsolateral prefrontal cortex, a crucial cerebral region linked to numerous advanced cognitive operations, was chosen for fNIRS monitoring. This region comprised seven channels on the left and right sides, resulting in 14 channels. According to the four types of changes in the relationship between the main components of the Oxy-Hb and Deoxy-Hb curves during the execution phase of the plantar contact task, A + B (Type A and B in Figure 6) was considered activated, and C + D (Type C and D in Figure 6) was the inactive state. The number of each curve type is shown in Tables 1, 2. 64 dorsolateral prefrontal channels were activated, 76 were inactivated in the paralyzed side, 33 dorsolateral prefrontal channels were activated, and 107 were inactivated in the healthy side during the open-eye and closed-eye conditions, respectively. In the closed-eye condition, the paralyzed side performed the task with 47 activated and 93 inactive dorsolateral prefrontal channels and the healthy side performed the task with 50 activated and 90 inactive dorsolateral prefrontal channels.

**Table 1:** Number of each curve type for the relationship between Oxy-Hb and Deoxy-Hb in the dorsolateral prefrontal brain region under the eyes-open condition.

	Paralysis side		Healthy side	
Curve type	Left dorsolateral prefrontal	Right dorsolateral prefrontal	Left dorsolateral prefrontal	Right dorsolateral prefrontal
A	4	6	0	0
B	24	30	22	11
C	34	24	17	36
D	8	10	31	23

**Table 2:** Number of each curve type for the relationship between Oxy-Hb and Deoxy-Hb in dorsolateral prefrontal brain regions under closed-eye condition.

	Paralysis side		Healthy side	
Curve type	Left dorsolateral prefrontal	Right dorsolateral prefrontal	Left dorsolateral prefrontal	Right dorsolateral prefrontal
A	12	15	7	18
B	13	7	20	5
C	18	14	33	17
D	27	34	10	30

### 3.2. Effect of open and closed-eye conditions on paralyzed versus healthy side performing the task

The stratified chi-square test was employed to compute the chi-square test results separately for the aggregate data under both open and closed eyes conditions (Table 3). The Test of Homogeneity of Odds Ratio (OR), utilized for determining the consistency of OR values across varying strata, is also called the test of homogeneity of OR values. The statistics associated with these two homogeneity tests and their test results are documented in Table 4. For the Breslow-Day method, χ^2^ was 9.259 with *p* = 0.002, while for the Tarone method, χ^2^ was 9.258 with *p* = 0.002. Both methodologies exhibited *p* < 0.05, which implies that post-stratification, according to whether the eyes were open or closed, there exists a degree of heterogeneity in the OR values between strata. Combining OR values at this time is inappropriate, and stratification is recommended for reporting.

**Table 3:** Pearson chi-square test results.

Item	χ^2^	*p*
Closed-eye conditions	0.142	0.706
Eyes-open condition	15.15	0.000

**Table 4:** Odds ratio homogeneity test.

Methods	χ^2^	*p*
Breslow-Day	9.259	0.002
Tarone’s	9.258	0.002

In the closed-eye condition, performing the plantar task on either the paralyzed or healthy side showed no significant effect on dorsolateral prefrontal activation (Pearson χ2 = 0.142, *p* = 0.706). In the eyes-open condition, we found a statistically significant difference (Pearson χ2 = 15.15, *p* = 0.002) in dorsolateral prefrontal lobe activation between the paralyzed and healthy sides, suggesting that the side on which the plantar task is performed influences activation.

### 3.3. Activation of the dorsolateral prefrontal lobe in the eyes-open condition with paralysis versus the healthy side performing the task

The previous results indicated that the eyes-open condition was influential in the execution of the plantar contact task on the paralyzed side versus the healthy side. Therefore, after removing the eyes-open and eyes-closed factors, the relationship between the paralyzed side versus the healthy side in performing the task and the activation of the dorsolateral prefrontal lobe was tested using both Cochran’s and Mantel–Haenszel chi-square tests, the former being a modified version of the latter, and the results are shown in Table 5. *p*-values were found to be less than 0.05, indicating that the paralyzed side of the patient versus the healthy side differed in activation of the dorsolateral prefrontal lobe when performing the plantar contact task.

**Table 5:** Independence test of the conditions.

Methods	χ^2^	*p*
Cochran’s	6.183	0.013
Mantel–Haenszel	5.729	0.017

The Mantel–Haenszel odds ratio was estimated to be 0.646. In this study, the variables were set as follows: for ‘side’ (1 = paralyzed, 2 = healthy) and for ‘activation’ (1 = unactivated/C + D, 2 = activated/A + B). Hence, an OR of 0.646 indicates that dorsolateral prefrontal activation was 0.646 times higher on the healthy side than the paralyzed one during task performance. In other words, the dorsolateral prefrontal activation during task performance on the paralyzed side was 1.55 times greater than on the healthy side. In the eyes-open condition, the paralyzed side was more likely to activate the dorsolateral prefrontal during the plantar contact task.

## 4. Discussions

The role of cognitive function in motor tasks has gained increasing attention in research, and the significance of cortical function in the neuromodulatory mechanisms associated with walking has been highlighted. In this study, we examined the impact of a plantar contact task on the activation of the dorsolateral prefrontal cortex in patients with post-stroke hemiplegia under both eyes-open and eyes-closed conditions. The patients carried out the task at a self-determined pace, sequentially transitioning the ball from the heel to the toe by flexing and extending the knee joint and continuously experiencing the trajectory change of the ball on the foot sole. Based on the fNIRS observations, the execution of the plantar contact task was effective in activating the dorsolateral prefrontal lobe. This task requires sensory input to the plantar surface of the foot regarding the blob’s trajectory and movement, generating a perceptual experience. Patients in a stable sitting posture primarily innervated the knee and ankle joints of the lower limbs to control the small ball planetary, signifying substantial cognitive engagement in this motor process.

Cortical afferents to the frontal motor cortex come from three sources: parietal somatosensory cortex, prefrontal cortex, and cingulate cortex (Fuster, 1993; Yip and Lui, 2023). In contrast, previous studies in rhesus monkeys have shown that the prefrontal cortex, primarily responsible for cognitive functions, has interactive fiber connections with the optic cortex (striate area), temporal lobe, and parietal lobe. It has direct or indirect fiber connections with the basal forebrain, cingulate gyrus, and hippocampus, extending fiber projections to the basal ganglia and hypothalamus. The prefrontal cortex has a well-developed granular layer IV that receives direct projections from the dorsomedial thalamic nucleus, the only neocortex with interactive fiber connections to this cortical information “portal. It is the only neocortex that interacts with this cortical information “portal.” This complex fiber connection pattern enables it to play a key role in perceiving abstract rules, working memory, attentional regulation, and cognitive functions such as planning and strategy of behavior, thinking, and reasoning (Kandel et al., 2000). Radiological studies have shown that cognitive task training combined with motor training enhances the hemodynamics in the dorsolateral prefrontal cortex (Erickson et al., 2007). Watanabe and Funahashi (2018) suggested that the parallel processing of multitask information mainly occurs due to the involvement of the dorsolateral prefrontal lobe, selectively activating different prefrontal cortex regions when processing various task modules. Stroke patients demonstrated enhanced activation of brain regions in the superior frontal gyrus, inferior frontal gyrus, bilateral cingulate gyrus, and right precentral gyrus associated with motor performance and learning, somatosensory, motor planning, and conflict information processing (Peters et al., 2019).

We also observed variability in dorsolateral prefrontal lobe activation related to whether the eyes were open or closed. After considering the stratified influences of open and closed eyes, in the closed-eye situation, the execution of the plantar contact task on the paralyzed side or healthy side did not affect dorsolateral prefrontal activation. In contrast, in the eyes-open condition, the execution of the plantar contact task on the paralyzed side or the healthy side was an important influence on dorsolateral prefrontal activation, and the dorsolateral prefrontal activation was 1.55 times higher on the paralyzed side than on the healthy side during the task. It is suggested that the change in motor control difficulty associated with performing the task on the paralyzed or healthy side with eyes open and external visual influences can enhance the activation of the cognitive control cortex represented by the dorsolateral prefrontal lobe. Studies have also probed motor cortical excitability by open versus closed eyes and similarly found that the stimulus–response curve obtained with eyes open was steeper than that obtained with eyes closed and that the closed-eye state may affect the recruitment of cortical circuits and thus reduce evoked motor output, which is also consistent with our results (Chen and Huang, 2018). In the eyes-open condition, the paralyzed side of the task is more activated to the dorsolateral prefrontal lobe and less activated to the healthy side. Compared to the healthy side, the increased difficulty of performing the plantar task on the paralyzed side necessitates greater activation of the cognitive control cortex. This allows for more robust information input to the regions of the brain processing the stimulus. Moreover, increasing the complexity of cognitive-motor tasks enhances the activation and interconnectedness of cortical networks (Rietschel et al., 2012). The plantar contact task involves visual conduction as well as proprioception in the eyes-open condition and proprioceptive conduction in the eyes-closed condition. It has been found that motor and cognitive tasks compete with each other for attentional resources and reorganize their allocation when a person is completing a cognitive-motor task. Allocating a portion of the resources fully devoted to cognition and proprioception to vision when eyes are open, the effective attentional resources allocated to the proprioceptive task are reduced (Snijders et al., 2007). Some scholars have argued (Walker et al., 2000; Ruffieux et al., 2015) that the visual system ensures the stability of the body by collecting information about its spatial position and other information, and that plantar tasks can be better accomplished. When the visual input is blocked when the eyes are closed, the visual feedback will be reduced, and the function of the vestibular system and proprioceptive system of the human body will be reduced. As a result, the human body’s postural adjustment ability decreases when performing tasks with eyes closed compared to eyes open, making it more difficult to complete the task. Some scholars have also found (Remaud et al., 2012; Kabbaligere et al., 2017) that visual, vestibular and cognitive functions are all important factors in the control of human position and movement. When a dual task is performed with normal visual input, the visual center will compensate for the completion of the cognitive task. It has also been found that brain activity recorded by electroencephalogram (EEG) is strongly modulated by the closed-eye state compared to the open-eye state. The use of fNIRS to measure cortical activity implies that a decrease in activity may reflect reduced utilization of a brain region (Lustig et al., 2009; Cabeza et al., 2018), whereas an increase in activity may signal a compensatory recruitment mechanism (Cabeza et al., 2002; Schneider-Garces et al., 2010). The higher the task difficulty, the greater the need for mobilization and recruitment within the compensable range for motor control, resulting in stronger activation of cognitive control brain regions (Qiao et al., 2020).

Interestingly, no type A curves were observed during the execution of the plantar contact task on the healthy side, with type A representing typical activation curve changes. This suggests that less difficult conditions are less likely to evoke a substantial blood oxygen response in the prefrontal lobe, leading to a typical activation pattern. Information from the occipital and parietal lobes related to vision and somatosensation forms perceptions translated into actual actions (motor output), primarily via the dorsolateral prefrontal cortex, which directly connects with the motor cortex (Miller and Cohen, 2001; Wallis et al., 2001; Szczepanski and Knight, 2014). This is consistent with earlier theories proposing a hierarchical organization in the prefrontal cortex, with the anterior end controlling abstract cognitive abilities and the posterior end regulating motor functions (Badre and D'esposito, 2009). However, it has also been suggested that the frontopolar and medial prefrontal cortex are closely related to the limbic system of the medial temporal lobe (Medial temporal) and thus have a stronger role in long-term memory, emotion, and motivation. Although various subdivisions of the prefrontal cortex control different aspects of cognitive function and play a major role in a specific function, they operate in concert when performing a specific task (Erickson et al., 2007). The most important neuromodulatory function performed by the dorsolateral prefrontal lobe is related to the inhibitory effect of this region on the amygdala and the hypothalamic–pituitary–adrenal axis. It usually relies on connections with other subcortical areas, which are then translated into action motivation via the limbic and mesocortical dopamine systems (Bigliassi and Filho, 2022). As stroke patients recover, external stimuli trigger endogenous neural repair mechanisms. These mechanisms are stimulated by motor training and cognitive behaviors, which together promote cortical activation. It promotes neural regeneration and increases the number of cortical neuronal synapses, which leads to faster information processing (Pang et al., 2018).

## 5. Conclusion

The execution of the plantar contact task, as observed through fNIRS, can effectively stimulate the dorsolateral prefrontal lobe. When combined with conditions of open and closed eyes, this activation effect can be enhanced by implementing the plantar task through the paralyzed side of the patient and moderately increasing task difficulty. Therapies rooted in such cognitive-motor techniques may provide a novel approach to promote motor function recovery through the perception and stimulation of cognitive control brain regions; however, their therapeutic impacts warrant further exploration. This study has certain limitations as it focused solely on the channels encompassing the dorsolateral prefrontal brain region and included a small patient population suffering from cerebral infarction.

## Data availability statement

The raw data supporting the conclusions of this article will be made available by the authors, without undue reservation.

## Ethics statement

The studies involving humans were approved by Ethics Committee of Beijing Rehabilitation Hospital, Capital Medical University. The studies were conducted in accordance with the local legislation and institutional requirements. The participants provided their written informed consent to participate in this study.

## Author contributions

Z-QY: Methodology, Writing – original draft. M-FW: Data curation, Writing – original draft. J-NX: Funding acquisition, Writing – review and editing.

## Conflict of interest

The authors declare that the research was conducted in the absence of any commercial or financial relationships that could be construed as a potential conflict of interest.

## Publisher’s note

All claims expressed in this article are solely those of the authors and do not necessarily represent those of their affiliated organizations, or those of the publisher, the editors and the reviewers. Any product that may be evaluated in this article, or claim that may be made by its manufacturer, is not guaranteed or endorsed by the publisher.

## Abbreviations

fNIRS: Functional near-infrared spectroscopy
ADL: Activities of daily living
Oxy-Hb: Oxygenated hemoglobin
Deoxy-Hb: Deoxygenated hemoglobin
NIR: near-Infrared
PFC: Prefrontal cortex
Fp: Frontopolar midline point
ROIs: Regions of interest
RDPFC: Right dorsolateral prefrontal cortex
LDPFC: Left dorsolateral prefrontal cortex
OR: Odds ratio
